# Time trends in limited lung function among German middle-aged and older adults

**DOI:** 10.1038/s41598-024-55624-2

**Published:** 2024-02-29

**Authors:** Johannes Beller, Batoul Safieddine, Stefanie Sperlich, Juliane Tetzlaff, Siegfried Geyer

**Affiliations:** Hannover Medical School, Center for Public Health and Health Care, Medical Sociology Unit, Carl-Neuberg-Str. 1, 30625 Hannover, Germany

**Keywords:** Public health, Epidemiology

## Abstract

Limited lung function represents a serious health impairment. However, studies investigating changes in limited lung function over time are rare. Thus, the current study investigates time-related changes in limited lung function and potential social inequalities. Data from the 2008 and 2017 waves of the population-based German Aging Survey were used in a repeated cross-sectional study design (*N* = 8778), including participants aged 40 years and older. Lung function was assessed by the peak flow test. Socio-economic indicators included educational attainment, income and occupational group. Additionally, smoking history, occupational exposure to fumes and gases, and physical exercise were used as potentially explanatory variables for the observed changes. We found that the prevalence of limited lung function decreased strongly over time on a descriptive level from 9.0 to 5.4%. In line with these results, a decreasing trend emerged (OR = 0.48) when controlling for age and gender differences. When additionally controlling for changes in socio-economic indicators and explanatory variables there were still significant decreases over time, but the decline was slightly reduced (OR = 0.57). Moreover, similar significant relative decreases over time occurred for middle-aged and older participants, female and male participants, and those belonging to the different socio-economic groups. Thus, limited lung function generally decreased over time. This decrease could partially be explained by beneficial developments in socio-economic indicators, smoking, occupational exposures, and physical exercise. Future studies might investigate how changes in medicinal treatment and prevention efforts have contributed to the observed beneficial trends in lung health.

## Introduction

### Changes and inequalities in limited lung function over time: less reduced peak flow for some but not all?

Lung function is an essential measure of overall health^1–3^. Lung functioning reflects the efficiency of the lungs in exchanging oxygen and carbon dioxide. As such, good lung function is seen as especially important for maintaining good cardiovascular health^4^. A decline in lung function can be caused by a variety of factors, including exposure to pollution, smoking, sedentary lifestyle, and chronic respiratory conditions such as asthma or chronic obstructive pulmonary disease^5–8^. Even a moderately limited lung function has been shown to increase mortality risk^1^. As such, lung functioning is often used as the central endpoint in clinical trials^9,10^.

### Trends in limited lung function

Few studies have estimated time trends in limited lung functioning in the general population, with mixed results: Among those studies reporting an increase in limited lung function, Fors and Thorslund^11^ explored trends and educational disparities in a range of health outcomes among the oldest old (those aged 80 and older) in Sweden with data from 1992, 2002 and 2011. The results showed increases in limited peak flow over time, which constitutes a popular measure of limited lung functioning. At the same time, the authors found that educational disparities in their health measures remained largely unaffected to the disadvantage of those with lower educational attainment. Similar increases in limited lung function were for example found by West and colleagues^12^ and Maio and colleagues^13^.

As a contrary finding, Lak and colleagues^14^ compared lung function in two cohorts of 75 year-olds in Sweden, one who was 75 years old in 1976–1977 and one who was 75 years old in 2005–2006. The study found that the mean peak expiratory flow was higher in the later-born cohort of 75 year-olds, as compared to those examined in 1976–1977, suggesting improved lung functioning over time. This increase could partially be explained by improvements in factors such as smoking and physical activity. However, similar studies also reported improved lung function over time^15,16^. Thus, the literature remains unclear regarding trends in lung functioning.

The interpretation of these results is further complicated by the varying trends of proximate factors for lung functioning: For example, if there are changes in the rates of smoking or exposure to air pollution, this could impact the prevalence of limited lung function. In countries like Germany, smoking prevalence has been reported to be declining, particularly in younger age groups, but tobacco use remains a highly prevalent public health problem^17^. Additionally, changes in the prevalence of conditions that directly lead to limited lung function, such as asthma, pneumonia and chronic obstructive pulmonary disease might impact the prevalence of limited lung function. In the case of chronic obstructive pulmonary disease, prevalence has been shown to have increased in female participants but decreased in male participants in 28 European countries between 2001 and 2019, with similar results in lung cancer^18,19^. Furthermore, medical advances and improvements in healthcare may help to prevent or slow the progression of limited lung function, which could also affect its prevalence^20^. Finally, changes in the overall population's health and lifestyle factors, such as diet and exercise, could also impact changes of limited lung function over time. In this case several studies have pointed to increases in morbidity, especially among middle-aged adults, as well as declines in morbidity, especially among older adults, which might lead to mixed trends in limited lung function over time^21–27^. Similarly, while frequency of physical exercise has increased over time, sedentary lifestyle such as prolonged sitting has been reported to has increased as well such that trends in limited lung function remain unclear^28^.

### Aim of the study

In summary, given the importance of lung functioning to healthy ageing and given that there are mixed results regarding population-based changes over time in limited lung function, further studies on trends in lung functioning are needed. It seems especially important to study changes in limited lung function over a broader age-range as most previous studies had focused on the oldest old or older participations. Additionally, as has been extensively reported in the literature on social inequalities, there might be large socio-economic differences in trends in limited lung function that might explain the diversity of the results found. Therefore, the current study contributes to the literature by examining trends in limited lung function in a population-based sample of middle-aged and older adults and by analysing how these trends differ according to socio-economic indicators. To the knowledge of the authors, the current study is the first one to empirically examine trends in limited lung functioning and their socio-economic differences in Germany.

## Methods

### Sample

The data for this study were drawn from the 2008 wave and 2017 wave of the German Aging Survey (DEAS), which is a population-based study of Germans aged 40 and older. The DEAS is provided by the Research Data Center of the German Center of Gerontology^29^. The DEAS has used probability sampling to randomly select new baseline participants in 1996, 2002, 2008 and 2014, and additionally re-contacted them in subsequent waves including 2002, 2008, 2011, 2014, and 2017. All interviews are conducted in-person at the participant's residence. Ethics board approval was not required for the current study, because only secondary data analysis of the completely anonymized data was conducted, and ethical approval is not mandatory for general surveys in Germany when anonymized data are analyzed. As such, the DEAS does not meet the criteria for requiring an ethical statement (risk for the respondents, lack of information about the aims of the study, examination of patients). This rationale is supported by the guidelines of the German Research Foundation available at https://www.dfg.de/en/research_funding/faq/faq_humanities_social_science/index.html. The German Ageing Survey meets the ethical standards delineated in the 1964 Declaration of Helsinki and its amendments. Prior to the interview, written informed consent was given by all participants of the study. The permanent advisory board of the German Centre of Gerontology, however, approved of the survey including its sampling method, the consent to participate and the instruments used in the study. For this study, data were used from all participants who consented to fill out a drop-off questionnaire and who consented to additional measurements including a peak flow meter in 2008 and 2017^30^. The 2008 wave was the first wave in which a lung function measurement was obtained, whereas 2017 was the last available wave in which a measure of lung function was obtained. Despite the samples consisting of first-time and repeat participants, only about 15% of all observations stem from additionally including observations in 2017 of participants that had already participated in 2008. Thus, the data essentially constitute a repeated cross-sectional study design by which changes in limited lung function over time can be investigated by comparing the prevalence of limited lung function of the 2008 sample with the prevalence of limited lung function of the 2017 sample. After excluding participants with missing values, a final sample of N = 8778 participants was used for the analysis.

### Measures

*Limited Lung Function.* In this study, lung function was measured using the peak flow method. The peak flow method is a commonly used technique for measuring lung function that involves using a peak flow meter, which measures the maximum speed at which a person can exhale in accordance with the standard procedure established by Nunn and Gregg^31^. The person being tested is asked to take a deep breath and exhale into the peak flow meter as hard and as fast as possible. Participants were asked to perform two measurements with a standardized peak flow meter (Mini-Wright) and the highest obtained value was recorded. Prior to measurement, participants were instructed on how to use the peak flow meter correctly and how to avoid common errors. Lung function testing was supervised by trained interviewers. The same device type was used for all participants and calibrated according to the manufacturer’s instructions. This method is generally considered to be a quick and simple way to measure lung function and is seen as especially useful for monitoring general lung function in large population-based studies^3,31–33^. Equations from European spirometry guidelines, which are standardized according to several factors such as age and gender, were used to calculate percentage of predicted lung function to identify participants with severely limited lung function^34^. Participants with peak expiratory flow rates of less than 50% of predicted were classified as having limited lung function, in line with previous studies^35^. Additional robustness analyses were conducted with peak expiratory flow rates of less than 40% and 60% of predicted.

*Covariates.* Socioeconomic status was measured using the three often used indicators education, income, and occupational group^36^. Education was measured by the highest obtained school leaving qualification in line with the International Standard Classification of Education (ISCED) as “Low” (ISCED levels 0–2), “Intermediate” (ISCED levels 3–4), or “High” (ISCED levels 5–6).

Income was based on the participants' self-report of their total monthly net household income in euros^29^. To improve comparability, the percentage of the equivalized income was calculated by adjusting the net income according to household size and then calculating the income relative to the mean equivalent income in the general German population. Income was classified into three groups: “Low” (income < 80% of the mean equivalent income in the population), “Intermediate” (income >  = 80% and <  = 120% of the mean equivalent income in the population), and “High” (income > 120% of the mean equivalent income in the population).

Occupational group was used as the occupation-related socioeconomic indicator. In this case, occupations were classified according to the International Standard Classification of Occupations (ISCO) as being high skilled white collar (WC-HS; ISCO major groups 1, 2 and 3), low skilled white collar (WC-LS; ISCO major groups 4 and 5), high skilled blue collar (BC-HS; ISCO major groups 6 and 7), or low skilled blue collar (BC-LS; ISCO major groups 8 and 9).

Information on smoking status was not identical between waves. However, one constant answer option between waves regarding one’s smoking behavior was to have never smoked. Thus, smoking status was operationalized in a harmonized way as “Never having smoked” (0) vs “Have smoked or I am currently smoking” (1). Job exposure to fumes and gases was operationalized via the 2006 (to operationalize job exposures in the first measurement point) and 2018 (to operationalize job exposures in the second measurement point) BIBB/BAuA Employment Surveys^37,38^ based on responses to the following question: Participants were asked to indicate how often they worked in the presence of smoke, dust, or gases, vapors. Occupational groups were classified according to third hierarchy level of the ISCO. Proportion of participants per occupational group that responded that they were at least “Often” exposed was used as the level of exposure indicator. Thus, values could vary from 0 (0% of participants of a specific occupational group reported being often exposed to smoke, dust, or gases, vapors) to 1 (100% of participants of a specific occupational group reported being often exposed smoke, dust, or gases, vapors). Additionally, physical exercise was operationalized by participant responses to a question regarding the amount of time they are “usually active in sports”. Responses were dichotomized as “Less than weekly physical exercise” (0) vs. “At least weekly physical exercise” (1). We also included information on the presence of three self-reported diagnoses of chronic health issues (“not reported to be diagnosed with” vs. “reported to be diagnosed with”): Chronic lung disease, diabetes, hypertension. Due to the unclear validity of these self-reported diagnoses, these results are only reported descriptively. Finally, age (in years) and (self-described) gender as male or female were included in the analyses.

### Data analysis

Chi-Squared and t-tests were conducted to provide basic descriptive statistics of changes in lung functioning between the 2008 and 2017 samples. Multiple logistic regression analyses were then used to examine the degree to which limited lung functioning changed between the samples of the two time points and whether these changes could be explained by the predictor variables. Two models with increasing complexity were calculated, starting with limited lung function predicted by time period, age, and gender (model 1) and then additionally adding education, occupation, smoking history, physical exercise, and job exposure to fumes and gases as predictors (model 2). Changes in the effect size of the regression coefficient for time period between models were used to identify the factors that contributed to changes in lung functioning over time^39^. By comparing the size of the effect of “time period” between the two different models with and without confounders controlled for, the study estimated the degree to which these confounders explained the observed differences in limited lung functioning between the samples of the two time points. Time trends in raw lung functioning values were also analyzed to support the conclusions in the article with the results being presented in the Online Appendix. Time trends were also investigated via a propensity score analysis to examine the robustness of our results. We created a matched sample of participants from the two time periods using nearest neighbor matching with a caliper of 0.2, as reported in the Online Appendix. As the last data analytical step, trend analyses were also stratified according to sociodemographic groups to examine the development of social inequalities over time. All regression analyses used the supplied weights. R (version 4.3.2) was used for all statistical analyses.

## Results

As depicted in Table 1, participants were on average 62.29 (range = 40–92; *SD* = 11.33) years old, with 48% being female. On average, 7.4% of participants were classified as having limited lung function. As also depicted in Table 1, participants with a limited lung function were older, more likely to have lower levels of educational attainment, to belong to lower income groups, and to work or have worked in blue collar occupations. Additionally, participants with limited lung function also were more strongly exposed to occupational exposures, less likely to have never smoked, and less likely to perform regular physical exercise.

**Table 1:** Descriptive statistics and descriptive differences between those with and without limited lung function.

	Stratified by Limited Lung Function		
	overall	Not Limited	Limited
N	8778	8130	648
Limited Lung Function (%)	7.4	0.0	100.0
Age (mean (SD))	63.29 (11.33)	62.82 (11.24)	69.21 (10.89)
Gender (% Female)	47.8	47.9	47.7
Education (%)			
High	41.0	41.8	31.2
Intermediate	52.8	52.5	56.3
Low	6.2	5.7	12.5
Income (%)			
> 120%	31.9	33.0	19.0
80–120%	36.5	36.5	36.9
< 80%	31.6	30.6	44.1
Occupation (%)			
WC-HS	53.5	54.6	39.4
WC-LS	21.6	21.5	22.5
BC-HS	13.6	13.3	17.9
BC-LS	11.3	10.6	20.2
Occupational Exposure (mean (SD))	0.12 (0.13)	0.12 (0.13)	0.16 (0.16)
Smoking History (% Never Smoked)	41.8	42.1	38.1
Physical Exercice (% Weekly)	51.5	52.9	33.3

WC-HS: White Collar High Skilled; WC-LS: White Collar Low Skilled; BC-HS: Blue Collar High Skilled; BC-LS: Blue Collar Low Skilled. Please note that some participants were counted two times if they participated in both the 2008 and 2017 wave.

### Trends and explanation of trends

Next, prevalence of limited lung function and predictor variables were compared between time periods. As can be seen in Table 2, limited lung function strongly decreased over time, from 9.0% to 5.4%. At the same time, age, educational status, income status also increased. Additionally, participants were more likely to work or have worked in White Collar High Skilled occupations and were less likely to have worked in Blue Collar occupations. Accordingly, participants were less likely to suffer from occupational exposure to smokes and fumes, were more likely to have never smoked and were more likely to regularly participate in physical exercise. Also, the prevalence of hypertension (from 37% in 2008 to 48% in 2017), diabetes (11% to 14%) and chronic lung disease (5% to 7%) increased among the study participants (not shown in the table).

**Table 2:** Differences in limited lung function and sample characteristics over time.

	Stratified by time period			
	Overall	2008	2017	p
N	8778	4878	3900	
Limited lung function (%)	7.4	9.0	5.4	< .001
Age (mean (SD))	63.29 (11.33)	61.15 (11.63)	65.97 (10.35)	< .001
Gender (% Female)	47.8	47.4	48.5	.313
Education (%)				< .001
High	41.0	36.6	46.5	
Intermediate	52.8	55.1	49.9	
Low	6.2	8.3	3.6	
Income (%)				< .001
> 120%	31.9	29.8	34.6	
80–120%	36.5	36.1	37.0	
< 80%	31.6	34.1	28.4	
Occupation (%)				< .001
WC-HS	53.5	48.3	60.0	
WC-LS	21.6	21.5	21.7	
BC-HS	13.6	15.3	11.5	
BC-LS	11.3	14.8	6.8	
Occupational Exposure (mean (SD))	0.12 (0.13)	0.13 (0.14)	0.10 (0.13)	< .001
Smoking History (% Never Smoked)	41.8	37.8	46.7	< .001
Physical Exercice (% Weekly)	51.5	47.0	57.0	< .001

WC-HS: White Collar High Skilled; WC-LS: White Collar Low Skilled; BC-HS: Blue Collar High Skilled; BC-LS: Blue Collar Low Skilled. Please note that some participants were counted two times if they participated in both the 2008 and 2017 wave.

Next, logistic regression analyses were used to study trends in limited lung function. As can be seen in Table 3, there was a substantial effect of time period (OR = 0.48), suggesting that odds of having limited lung function strongly decreased between time periods. Similar results were obtained when using an alternative cut-off of peak expiratory flow rates of less than 60% of predicted (OR = 0.49), when using an alternative cut-off of peak expiratory flow rates of less than 40% of predicted (OR = 0.45), when restricting our sample in such a way that only those respondents who participated in only one of the surveys are included (OR = 0.51), and when using a matched sample (OR = 0.57). When controlling for the potentially explanatory variables (education, income, occupational group, occupational exposure, physical exercise), a significant but slightly reduced effect of time period was still found similar to the matched analysis (OR = 0.57). Thus, when including socio-economic and risk factor developments over time, about 17% of relative decrease over time could be explained by including relevant socio-demographic and behavioral risk factors.

**Table 3:** Explaining differences in limited lung function over time.

Predictors	OR	95%-CI	z	p	XXX	OR	95%-CI	z	p
Time Period	0.48	[0.40; 0.59]	-7.36	< .001		0.57	[0.47; 0.69]	-5.54	< .001
Age	1.90	[1.74; 2.07]	14.22	< .001		1.84	[1.68; 2.01]	13.09	< .001
Gender									
Male (Ref.)									
Female	1.11	[0.92; 1.32]	1.09	.278		1.13	[0.92; 1.39]	1.18	.239
Smoking History									
Has smoked (Ref.)									
Has never smoked						0.72	[0.59; 0.88]	-3.21	.001
Occupational Exposure						1.15	[0.46; 2.86]	0.29	.773
Physical Exercise						0.56	[0.46; 0.68]	-5.76	< .001
Educational Level									
High (Ref.)									
Intermediate						1.07	[0.84; 1.37]	0.56	.573
Low						1.23	[0.85; 1.79]	1.12	.261
Income									
> 120% (Ref.)									
80–120%						1.17	[0.91; 1.50]	1.24	.215
< 80%						1.38	[1.06; 1.78]	2.37	.018
Occupation									
WC-HS (Ref.)									
WC-LS						1.25	[0.95; 1.61]	1.61	.106
BC-HS						1.16	[0.79; 1.73]	0.76	.447
BC-LS						1.79	[1.28; 2.52]	3.40	< .001

OR: Odds Ratio; CI: Confidence Interval; z: z-value; p: p-value; WC-HS: White Collar High Skilled; WC-LS: White Collar Low Skilled; BC-HS: Blue Collar High Skilled; BC-LS: Blue Collar Low Skilled.

### Socioeconomic differences in trends

**Figure 1:**
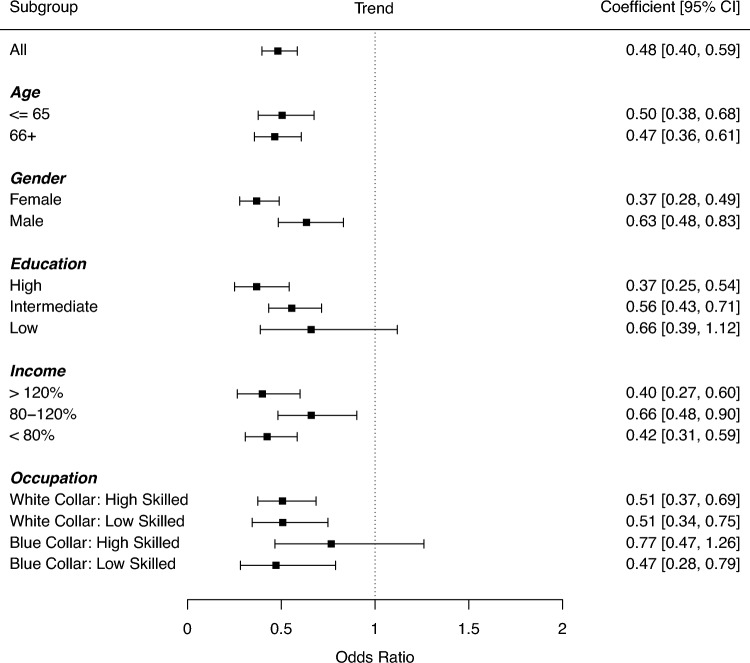
Socio-economic differences in changes in limited lung function over time. *Notes* CI: Confidence Interval.

As the final data analytical step, trends in limited lung function were analysed in different socio-economic groups, to examine the generalizability of the observed decreases. As displayed in Fig. 1, decreasing trends over time were found in all analysed substrata of the population, including middle-aged and older adults, female and male participants, those with higher, intermediate and low educational status, those with higher intermediate and lower income, and those from different occupational backgrounds. Only the trends regarding those with a lower educational attainment and those from Blue Collar High Skilled occupations were not statistically significant; in all other subgroups statistically significant decreases over time were observed.

## Discussion

Given the importance of lung function to healthy aging and morbidity development, we examined trends and socioeconomic differences in limited lung function. We found that the prevalence of limited lung function strongly decreased over time, from 9.0 to 5.4%. When controlling for important covariates, a decreasing trend was still observed. This decrease appeared in a similar way in most analysed socio-demographic groups, including in middle-aged and older participants, female and male participants, and those belonging to different education, income and occupational groups. Thus, limited lung function generally decreased over time in all subgroups considered, which was at least partly due to the beneficial developments in socio-economic indicators, smoking, occupational exposures, and physical exercise.

### Comparison with previous studies

These findings are in line with previous studies that have also observed a decline in limited lung function over time^14–16^ These studies, along with our own, suggest that limited lung function is decreasing over time. While our findings are in line with some previous studies, they also contradict others that have observed an increase in limited lung function over time. For example, one study found increases in limited peak flow over time in Sweden^11^. Similarly, another study found increases in the prevalence of many respiratory symptoms and diseases in Italy^13^. Discrepancies between studies could be due to differences in study design, population characteristics, or other factors and potential explanations as to the concrete reasons would be speculative at best. However, these conflicting findings highlight the need for further research to better understand the changes in limited lung function over time and their antecedents.

### Potential explanations for decreases in limited lung function

There are several potential explanations for the observed decrease in limited lung function over time, some of which had been examined empirically within the current study. One possible explanation is that improvements in socio-economic indicators, such as educational attainment and income, may have contributed to the decline in limited lung function. Higher levels of education and income have been associated with better health outcomes, including better lung health^36,40^. Additionally, smoking, occupational exposures and physical exercise have all been shown to improve lung function and have also been shown to have changed favourably over time in the current study. Consequently, a substantive part of the observed relative decreases in limited lung function over time could be explained once these explanatory factors were considered.

On the other hand, the current study did not focus on the potential effects of another important macro trend: the advances in medical diagnoses, treatments and medications. However, it seems highly likely that these factors may have had an impact on observed trends^41–43^. Perhaps improvements to medical technology and diagnostic accuracy led to earlier identification and treatment of respiratory conditions which could then contribute to better outcomes over time at the population level. For example, new treatment regimens involving inhaled corticosteroids used for asthma or other chronic lung issues, long-acting bronchodilators for COPD, and antibiotics for treating bacterial lung infections all potentially contributed to improved treatment outcomes for those affected by pulmonary problems.

Future research should investigate the potential role of advances in medical diagnoses, treatments, and medications in the observed decline in limited lung function. This could include examining changes in the accuracy and availability of diagnostic tests, as well as changes in the types and effectiveness of treatments and medications for lung conditions. Additionally, research could explore whether changes in access to medical care, such as increased availability of specialized care, have contributed to the observed decline in limited lung function.

### Implications

The findings of this study have implications for the theories of morbidity compression and morbidity expansion, which are two contrasting theoretical perspectives on how healthy aging will change over time. While the theory of morbidity expansion suggests that medical advances and increasing life expectancy will result in an increase in the time spent with illness and disability^44^, the theory of morbidity compression posits that improvements in healthy lifestyles and prevention of chronic diseases will result in a reduction in the amount of time spent with illness and disability^45^. The findings of this study, which showed a decline in limited lung function over time in middle-aged and older adults, are more consistent with the theory of morbidity compression. This is opposed to some other studies which suggested increases in morbidity, especially regarding activity limitations, among middle-aged adults^22,46^. Also, some previous studies regarding lung diseases found varying differential trends in some subgroups^19,47^. Accordingly, further research is needed before firm conclusions regarding a possible expansion or compression of morbidity regarding lung health can be made.

From a practical perspective, the stark decrease of limited lung function over time provides a positive outlook. Pulmonary diseases and limited functioning constitute one of the leading causes of morbidity and mortality in Germany^48^. Decreases in limited lung function thus constitute one major beneficial health trend. It must be noted, however that this positive development in lung health is contrasted by several concerning trends in cardiovascular, mental and functional health, which have been observed primarily in middle-aged adults^21,49–51^. Thus, further studies are needed that analyse health trends.

### Limitations

The results of the current study are subject to limitations. Most importantly, peak flow represents only one specific indicator of lung functioning that is favourably used in large epidemiological studies due its ease of measurement^1,2^. While peak flow strongly correlates with other indicators of lung functioning and pulmonary disease severity, it cannot be used interchangeably with other indicators like the forced expiratory volume and forced vital capacity, especially in clinical diagnosis^32,33,52^. Similarly, there is a lack of well-validated predicted values for peak flow in the literature. For example, the reference values that we used to define limited lung function are assumed to apply to Europeans aged 18–70 years and thus may not be fully compatible with our sample of Germans aged 40–92 years old^34^. To examine the robustness of our findings, we thus repeated the analysis with different cut-off values for limited lung function, namely 40% and 60% of the predicted values. The results showed that the main conclusions of our study remained unchanged, regardless of the cut-off value used. The prevalence of limited lung function decreased strongly over time. However, future research should still aim to develop and validate predicted values for peak flow that are based on larger and more representative samples of the population.

Furthermore, the sample did not include institutionalized older adults and thus likely underestimates the true level of limited lung function in the population. One might argue that the differential mortality and institutionalization could have affected the changes in limited lung function over time. However, we think that this is unlikely to explain the observed decline in our study, because we used a population-based sample, we applied appropriate weights to account for selection bias, and we observed a similar decline in limited lung function among older and middle-aged adults, who are less likely to die or be institutionalized than older adults. Similarly, the samples included both baseline and repeat respondents, thus potentially having sample selectivity bias, especially because previous studies have shown biases in survey samples^53,54^. Additionally, the study only analysed changes between 2008 and 2017, which may not be sufficient to fully understand changes in limited lung function over time. The observed decline in limited lung function may be due to short-term changes, which might not translate to long-term trends. Observing longer time periods would be needed to better understand long-term changes in limited lung function. Finally, besides the objective measurement of lung functioning, the study relied on self-reported data, which may be subject to self-report bias and inaccuracies. Similarly, the lack of detailed information on respiratory morbidities and their treatment in the data might have limited the possibility to explain the trends in lung health in greater detail. Thus, more studies are needed to investigate trends and social inequalities in lung functioning using population-based data.

## Conclusion

In conclusion, the current study investigated changes in limited lung function from 2008 to 2017 and potential inequalities. The results showed that the prevalence of limited lung function decreased strongly over time, and similar declines were observed in middle-aged and older participants, female and male participants, and those belonging to different socio-economic groups. Thus, limited lung function appears to have generally decreased over time, at least partly due to beneficial developments in socio-economic indicators, smoking, occupational exposures, and physical exercise. However, before firm conclusions about trends in general lung health can be drawn, our results must be replicated. Additionally, further research is needed to better understand the biopsychosocial and medical factors that have contributed to the observed decline in limited lung function.

### Supplementary Information

Supplementary Information.

## Data Availability

The data are available via the Research Data Centres of the German Centre of Gerontology and the Federal Institute for Vocational Training and Education.
