# Supplementary material for: Time trends in limited lung function among German middle-aged and older adults
Source: Sci Rep. 2024 Feb 29;14:5036. doi: 10.1038/s41598-024-55624-2 (PMC10904379; doi:10.1038/s41598-024-55624-2)
Supplement: Supplementary file 1 — Supplementary Information. [file 41598_2024_55624_MOESM1_ESM.pdf]

# APPENDIX

To analyse the robustness of the obtained results changes in raw peak flow values were examined between 2008 and 2017. Here quantile regression was calculated which allows to estimate time trends across the whole distribution of the dependent variable. Thus, time trends in very low peak flow values can be calculated, which roughly corresponds to the definition of limited peak flow reported in the main manuscript. Additionally, time trends in median peak flow and time trends in very good peak flow values are also calculated.

Figure A1 displays the distribution of peak flow values in 2008 and 2017. As one can see, peak flow values appeared generally higher in 2017 as compared to 2008. This improvement appeared especially pronounced for the 5% quantile of the distribution. In contrast time trends were reversed for the 95% quantile, where a decrease was observed. Quantile regression results are displayed in Table A1. As one can see the 5% quantile of lung functioning, measured via peak flow significantly decreased over time, as did the 50% quantile. In contrast, there were no significant differences over time in the 95% quantile of lung functioning. Thus, limited and medium lung functioning values improved over time, but not very high lung functioning.

To additionally analyse the robustness of the obtained results changes in limited lung function were examined via comparing samples of the two time points matched for covariates. The matched samples are depicted in Table A2. As can be seen, descriptive statistics were very similar with respect to the distribution of covariates, but still differed substantially in terms of limited lung function (9.2% vs. 5.3%). When analyzing these changes via logistic regression analysis $OR = 0.57$, $p < .001$, resulted.

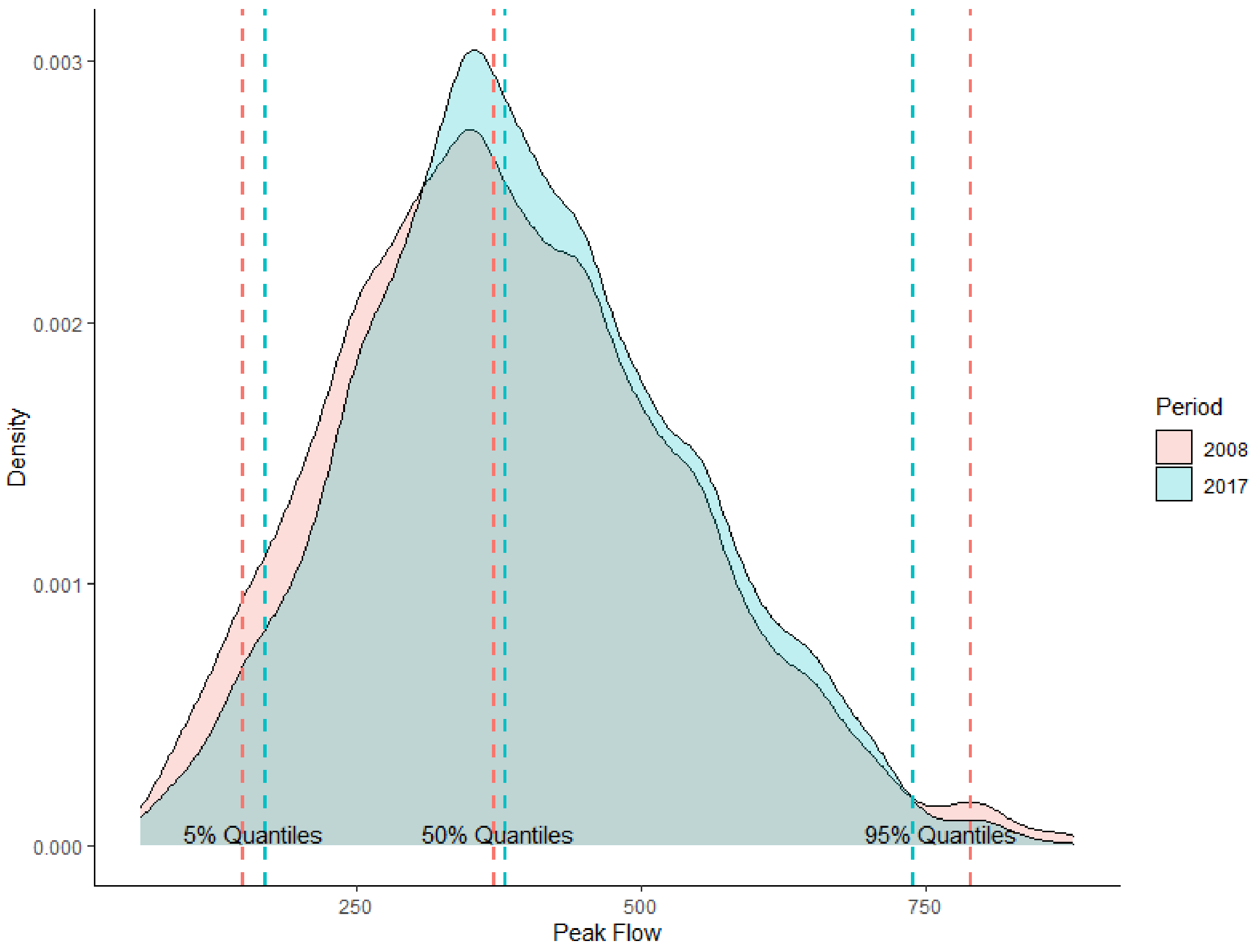


*Figure A1*. Raw peak flow values over time, with the 5%, 50% and 95% quantiles of the distributions.

*Table A1*. Quantile Regression Results.

| Predictor | *b* | *SE* | *t* | *p* |
|---|---|---|---|---|
| **5% Quantile** | | | | |
| Time Period (2017) | 35.26 | 3.80 | 9.29 | < .001 |
| Height | 2.81 | 0.30 | 9.27 | < .001 |
| Age | -3.86 | 0.17 | -22.55 | < .001 |
| Gender (Female) | -24.21 | 5.60 | -4.32 | < .001 |
| | | | | |
| **50% Quantile** | | | | |
| Time Period (2017) | 34.53 | 2.25 | 15.37 | < .001 |
| Height | 3.14 | 0.16 | 19.64 | < .001 |
| Age | -4.41 | 0.10 | -43.77 | < .001 |
| Gender (Female) | -111.35 | 3.07 | -36.28 | < .001 |
| | | | | |
| **95% Quantile** | | | | |
| Time Period (2017) | 2.93 | 5.11 | 0.57 | .567 |
| Height | 2.74 | 0.36 | 7.53 | < .001 |
| Age | -4.17 | 0.22 | -19.08 | < .001 |
| Gender (Female) | -176.36 | 6.64 | -26.54 | < .001 |

*Notes.* *b* = regression coefficient; *SE* = standard error; *t* = *t*-value; *p* = *p*-value

Table A2. Differences in limited lung function in matched samples over time.

| | Stratified by Time Period | | |
|---|---|---|---|
| | Overall | 2008 | 2017 |
| N | 7226 | 3613 | 3613 |
| Limited Lung Function (%) | 7.2 | 9.2 | 5.3 |
| Age (mean (SD)) | 64.55 (10.51) | 63.98 (10.81) | 65.13 (10.17) |
| Gender (% Female) | 48.3 | 48.0 | 48.5 |
| Education (%) | | | |
| High | 44.1 | 43.0 | 45.3 |
| Intermediate | 51.7 | 52.6 | 50.8 |
| Low | 4.1 | 4.4 | 3.9 |
| Income (%) | | | |
| > 120% | 34.1 | 34.0 | 34.3 |
| 80%-120% | 36.5 | 36.5 | 36.5 |
| < 80% | 29.4 | 29.5 | 29.2 |
| Occupation (%) | | | |
| WC-HS | 57.3 | 56.1 | 58.4 |
| WC-LS | 22.3 | 22.5 | 22.1 |
| BC-HS | 12.9 | 13.5 | 12.2 |
| BC-LS | 7.6 | 7.9 | 7.4 |
| Occupational Exposure (mean (SD)) | 0.11 (0.13) | 0.11 (0.12) | 0.11 (0.13) |
| Smoking History (% Never Smoked) | 44.4 | 43.3 | 45.4 |
| Physical Exercice (% Weekly) | 54.4 | 53.3 | 55.4 |

Notes. WC-HS: White Collar High Skilled; WC-LS: White Collar Low Skilled; BC-HS: Blue Collar High Skilled; BC-LS: Blue Collar Low Skilled. Please note that some participants were counted two times if they participated in both the 2008 and 2017 wave.
